# Refinement of primary central nervous system lymphoma prognostication and response assessment using 3-dimensional MRI

**DOI:** 10.1093/noajnl/vdaf090

**Published:** 2025-05-05

**Authors:** Jack O’Shaughnessy, Arina Martynchyk, Sze Ting Lee, Geoff Chong, Shivam Agrawal, Maciej Tatarczuch, Nariza Alysa Azryn, Gareth P Gregory, Leonid Churilov, Michael Wang, Colm Keane, Eliza A Hawkes

**Affiliations:** Olivia Newton John Cancer Research Institute at Austin Health, Australia; Olivia Newton John Cancer Research Institute at Austin Health, Australia; Austin Health, Molecular Imaging and therapy, Melbourne, Australia; University of Melbourne, Australia; Olivia Newton John Cancer Research Institute at Austin Health, Australia; Olivia Newton John Cancer Research Institute at Austin Health, Australia; School of Clinical Sciences at Monash Health, Faculty of Medicine, Nursing & Health Sciences, Monash University, Australia; Monash Health, Melbourne, Australia; Austin Health, Molecular Imaging and therapy, Melbourne, Australia; School of Clinical Sciences at Monash Health, Faculty of Medicine, Nursing & Health Sciences, Monash University, Australia; University of Melbourne, Australia; Austin Health, Molecular Imaging and therapy, Melbourne, Australia; Frazer Institute, University of Queensland, Australia; Princess Alexandra Hospital, Australia; School of Public Health and Preventive Medicine, Monash University, Melbourne, Australia; Olivia Newton John Cancer Research Institute at Austin Health, Australia

**Keywords:** 3-dimensional tumor volume, MRI, lymphoma, primary central nervous system lymphoma, PCNSL

## Abstract

**Background:**

Primary central nervous system lymphoma (PCNSL) is an aggressive lymphoma restricted to the CNS in which outcomes cannot be reliably predicted. The International PCNSL Collaborative Group developed standardized response assessment utilizing 2-dimensional (2D) Magnetic Resonance Imaging (MRI) tumor measurements. Considerable challenges of this approach exist due to many reasons. Recent glioblastoma and PCNSL data demonstrated that radiological assessment of baseline 3-dimensional volume (3DV) as well as 3DV reduction (3DVR) may be a sensitive prognostic parameter.

**Methods:**

Our multicentre retrospective study evaluated semiautomated 3DV in 74 PNCSL patients undergoing curative-intent chemoimmunotherapy.

**Results:**

Baseline tumor 3DV was not associated with survival. Compared to 3DVR < 58% (ROC-determined threshold based on our cohort), both interim and End-of-Treatment (EOT) 3DVR ≥ 58% in responding patients were associated with statistically significant prolonged 2-year progression-free survival (PFS) (interim: 73% (95%CI 57-83) versus 22% (95%CI 3-51), *P* = 0.005; EOT: 75% (95%CI 59-85) versus 0%, *P* = 0.002) and 2-year OS (interim: 83% (95%CI 68-91) versus 38% (95%CI 9-67), *P* = 0.02; EOT: 86% (95%CI 70-93) versus 0%, *P* = 0.0002). However, no significant differences in PFS or OS were observed in patients achieving standard 2D complete response (CR) compared to partial response (PR).

**Conclusion:**

Although PCNSL tumor 3DV at baseline is not associated with survival outcomes, 3DVR of ≥58% in interim and EOT confers superior PFS and OS. Whereas, no difference in survival was observed using standard 2D CR versus PR response assessment at the same time-points. 3DV calculations may offer a sensitive method of response assessment for PCNSL. We are currently validating this in clinical trials.

**Key points**

Interim and end of treatment 3-dimensional volumetric reduction ≥58% from baseline tumor volume in responding patients with primary central nervous system lymphoma is associated with longer survival in contrast to standard complete versus partial responses.

**Importance of the Study**

The current response evaluation criteria for primary central nervous system lymphoma (PCNSL) involves measuring the post-treatment change in cross sectional tumor area, however this does not reliably predict patient outcomes. Recent studies in solid brain tumors have shown that 3-dimensional volume (3DV) reduction may be a promising novel method for prognostication, but so far there is limited research applying this to PCNSL. The current study analyzed 193 MRIs from 74 patients from two tertiary centers, and demonstrated that patients with an interim or end of treatment volume reduction of 58% or greater had significantly longer survival. These results outperformed the predictions of existing response criteria, which was unable to distinguish the prognosis of patients with complete and partial response at these time-points. Our data indicates that measuring 3DV may function as an emerging biomarker that could potentially guide future treatment algorithms in trials for PCNSL.

Primary central nervous system lymphoma (PCNSL) is a rare, aggressive extranodal lymphoma limited to the brain, spinal cord, cerebrospinal fluid (CSF), and vitreoretinal compartment with an incidence of 0.5/100 000 per year.^1^ The most common histopathological subtype is diffuse-large B-cell lymphoma (DLBCL), accounting for 80% to 90% of PCNSL cases.^2,3^ PCNSL has a distinct molecular signature from systemic DLBCL which is consistent with significant differences in clinical behavior and outcomes.^3^ Despite diagnostic, staging and therapeutic advances with modern induction regimens incorporating high-dose methotrexate or thiotepa, PCNSL continues to portend a poor prognosis with a high rate of relapse.^2,4^ The median overall survival of PCNSL is approximately 20 months from the time of diagnosis with standard therapy,^5^ with 40% to 60% of patients experiencing disease progression or relapse despite often receiving intensive therapy.^6,7^ Disease staging is currently performed primarily with contrast-enhanced magnetic resonance imaging (CE-MRI). Dedicated CNS positron-emission tomography (PET) is not standard or validated for PCNSL but studies demonstrate potential additional diagnostic and prognostic information.^8^ Accurate prognostication is essential for informed decision-making and to potentially allow treatment escalation in those with early identification of high-risk disease but also de-escalation in those likely to do well. Clinical prognostic scores do exist, such as the International Extranodal Lymphoma Study Group (IELSG) experience which combines disease and patient factors known to be independently prognostic (age, performance status, lactate dehydrogenase (LDH) serum level, CSF protein concentration, deep brain structure involvement).^9^ However, a reliable tool combining radiographic features and clinical features for more accurate prognostication is lacking.

The prognostic utility of current PCNSL response assessment criteria is uncertain. Standard two-dimensional (2D) response criteria were developed by the International PCNSL Collaborative Group (IPCG) in 2005 using sum product measurements of CE-MRI that are performed at baseline and repeated after therapy to evaluate treatment response.^10^ In addition to radiological results, other parameters that are evaluated for CR assessment include eye examination, use of corticosteroids for lymphoma symptoms control and CSF analysis.^11^ Although this system remains the gold standard, limitations exist in delineating between complete response (CR) or unconfirmed complete response (CRu) and partial response (PR) or stable disease (SD) and defining outcomes according to these categories.

The IPCG recognized these potential limitations and was supportive of future studies to identify additional radiologic, functional, and laboratory that may be of value. Various clinical studies have subsequently shown that the response criteria are unreliable in differentiating outcomes between patients with PR and CR at multiple time-points. Kim et al. found no significant survival differences for these IPCG response groups at the EOT with high-dose methotrexate.^12^ These findings were replicated by Van der Meulen et al., who showed that the survival outcomes were not significantly different between EOT PR or CR status in the HOVON 105/ ALLG NHL 24 phase III clinical trial.^13^ Similarly, Tabouret et al. found highly variable outcomes for patients achieving PR,^14^ possibly suggesting that this is a heterogenous group and that further studies are required to elucidate which patients are at high risk for subsequent relapse or refractory disease.^13^ Therefore, there is a need to develop more reliable prognostication systems for PCNSL.

Using sophisticated techniques, 3-dimensional (3D) T1-weighted MRI subtraction maps were assessed in glioblastoma (GBM) patients and were demonstrated to be a robust biomarker in predicting OS.^11,15^ A subsequent GBM study reported that a volume reduction of >65% was an independently associated with improved overall survival (OS). Recently published analysis of patients with PCNSL by Lauer et al. reported an optimal cutoff of 97% tumor volume reduction.^16^ Systemic lymphoma measurements of total tumor volume and volume reductions—as measured on PET at baseline and after therapy—are predictive of outcomes independent of other risk factors across a large number of disease subtypes.^17^ However, volume measurements of PCNSL and the impact on outcomes are less well established.

Here, we describe baseline MRI 3D volumes (3DV), 3DV reduction (3DVR) measured using semiautomated methodology, standard 2D response assessment as outlined by IPCG and the association of results with 2-year progression-free (PFS) and OS for patients with treatment-naïve PCNSL treated with curative-intent chemoimmunotherapy (CIT).

## Methods

### Patient Selection

This was a multicentre observational analysis. Eligible patients included adults with a confirmed diagnosis of PCNSL between 2009 and 2021 treated with curative-intent CIT who had baseline, interim and post-treatment MRIs available for central analysis. Patients treated with palliative regimens (eg radiotherapy, corticosteroids alone) or with no identifiable MRI contrast-enhancing disease were excluded. Data on patient and disease characteristics, staging investigations, treatment details, and clinical outcomes were collected from hospital medical records at two specialized lymphoma centers. Data collection and transmission were conducted according to local regulations and this study was approved by the Austin Health Human Research Ethics Committee (LNR-17-Austin-18 as of 13.04.2017). All authors have access to primary data of analysis. All participants data were deidentified.

### MRI Evaluation

MRI scans were acquired at diagnosis (baseline MRI), after 2 to 5 cycles of CIT (Interim MRI) and end of treatment (EOT MRI). MRIs were performed according to IPCG recommendations and included contrast-enhanced T1w sequences pre- and postgadolinium contrast MRIs of 5 mm thickness. Standard 2D response assessment according to IPCG criteria^10^ was reported by board-certified radiologists at each time point. The participating centers had expert radiology assessment of the response at the time of reporting and the MRI reports were standardized using the IPCG response assessment criteria. These institutions also have routine lymphoma multidisciplinary meetings where cases are discussed. Thus, 2D responses were reported according to the standard IPCG criteria which incorporates CR as the complete resolution of all lymphoma lesions; partial response as a 50% or greater decrease in tumor size; progressive disease as at least a 25% increase in tumor size or the appearance of any new tumor lesion; and stable disease was defined as situations that did not meet any of these criteria. These were reconfirmed centrally by the expert MRI team undertaking the 3D reviews and correlated with the local reporting. Only in the event of a discrepancy was this reviewed further.

**Figure 2.**
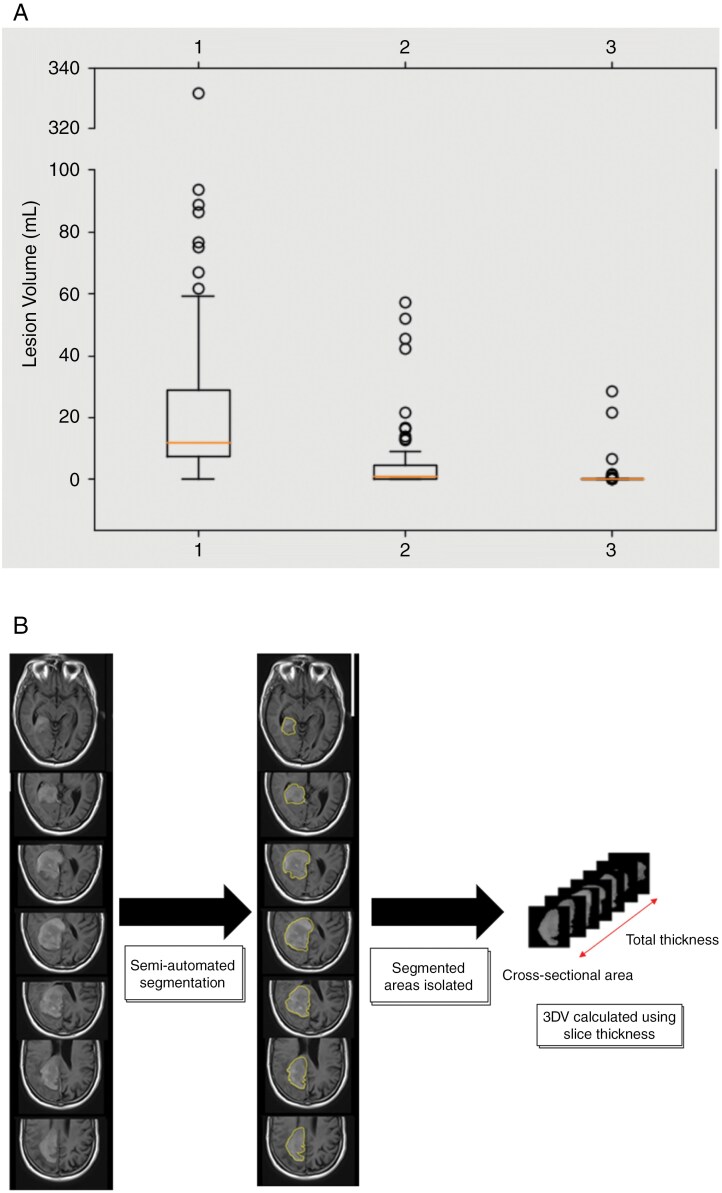
3-Dimentional Volume Data and Measurements. (A) Baseline (1), interim (2) and end of treatment (3) MRI volume data. (B) 3DV analysis.

Post-hoc 3DV analysis utilized segmentation of 3DV reconstructions using semiautomated software, MIM Maestro, was performed centrally, confirmed by two radiologists (Figure 2B). Volumetric contouring was performed on postgadolinium T1 weighted images (T1wGd) for all patients, with precontrast T1 and FLAIR sequences reviewed but not segmented. All enhancing disease on T1wGd were segmented and volumes recorded. Radiologist discretion was utilized based on the appearances over multiple time-points to ensure that only viable tumor was included. If precontrast images were not available for comparison, tumor volumes were calculated from other sequences such as diffusion‐weighted imaging with apparent diffusion coefficient and T2-weighted-fluid-attenuated inversion recovery images. All MRI contouring was performed on postcontrast scans.

### Statistical Analysis

Demographic and disease characteristics were summarized using descriptive statistics. Differences in baseline characteristics of patient subgroups were calculated using Fisher’s exact test using the following factors: age, gender, ECOG, LDH, IELSG risk categories^8^ and level of protein in CSF. To accommodate the sample size restrictions, instead of conducting individual univariate analyses for individual characteristics, a Cox regression analysis was performed on the validated IESLG risk categories that reflects the information presented by the combination of above variables.

Two-year PFS and OS were defined as clinical outcomes based on tumor biology and clinical rationale. PFS was defined as time from diagnosis to progression or death, and OS was calculated from time of diagnosis to death. Kaplan–Meier survival curve and log rank test were used for survival analysis and comparisons between patients with different baseline characteristics, 3DV thresholds and responses. For survival comparison the predefined published cut off of 65% for 3DVR for solid tumors was used.^11,15^ An optimal cutoff for 3DVR was also derived using a receiver operating curve (ROC) analysis of patient PFS and OS for this dataset.

All statistical analyses were performed using Stata Statistical Software v18, College Station, TX and R version 4.3.3 package “EZR.”^18^ Data collection and transmission were conducted according to local regulations and this study was approved by the Austin Health Human Research Ethics Committee (LNR-17-Austin-18 as of 13.04.2017). All authors have access to primary data of analysis.

We described the distribution of baseline 3DV, interim and EOT 3DVR. Subsequently, we compared PFS and OS using a median binary baseline and interim 3DV (i3DV) cutoff, baseline characteristics, interim 3DVR (i3DVR) and EOT 3DVR with different cut off as well as standard 2D response criteria at the interim and EOT response assessment.

## Results

### Baseline Characteristics

**Figure 1.**
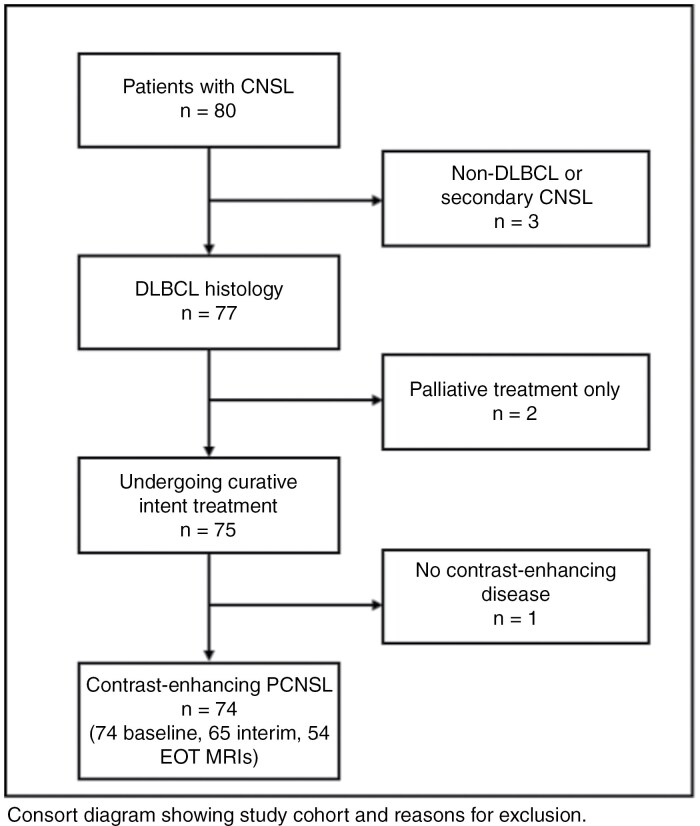
Patient Selection. CNSL, central nervous system lymphoma, DLBCL, diffuse-large B-cell lymphoma, PCNSL, primary central nervous system lymphoma.

Eighty patients were identified with biopsy-proven PCNSL. Of these, 74 were eligible (Figure 1). Patients with secondary CNS involvement, non-DLBCL histology, no baseline MRI or no evidence of contrast-enhancing disease were excluded. Median age was 67 years (range 22-86), 57% of patients were male. Baseline patient and disease characteristics, IELSG score risk categories, treatment details, interim, and EOT responses are summarized Table 1.

**Table 1.** Patients Baseline Characteristics and Outcomes

	Whole cohort	High 3DV (<11.8 ml)	Low 3DV (<11.8 ml)
Characteristic, *n*	74	36	38
Age (years) median, range	67 (22-86)		
>60 years, *n* (%)	50 (67)	24 (67)	26 (68)
			*P* > 0.99
			
Gender			
Male, *n* (%)	43 (57)	23 (64)	20 (53)
			*P* = 0.4
			
Follow up (months) median, range	44.2 (2-154)		
			
Performance status, *n* (%)			
ECOG 0-1	48 (65)	19 (53)	29 (76)
ECOG 2-4	26 (35)	17 (47)	9 (24)
			*P* = 0.051
			
LDH, n (%)			
Elevated	7 (10)	4 (11)	4 (11)
Normal	60 (78)	28 (78)	31 (82)
			*P* > 0.99
Not performed	9 (12)	4 (11)	3 (8)
			
CSF, *n* (%)			
Elevated	30 (41)	11 (31)	19 (50)
Normal	31 (42)	18 (50)	13 (34)
			*P* = 0.1
			
Not performed	13 (18)	7 (19)	6 (16)
			
Treatment type, *n* (%)			
R-MPV	41 (54)	23 (64)	19 (50)
MATRIX	10 (13)	6 (17)	4 (11)
Other	25 (33)	7 (19)	15 (39)
Consolidation with RT	17 (23)	9 (25)	8 (29)
Consolidation with ASCT	3 (4)	1 (3)	2 (5)
			
2D standard response (interim), *n* (%)			
PD	6 (8)	3 (8)	3 (8)
SD	1 (1)	0	1 (3)
PR	44 (59)	26 (72)	18 (47)
CR	21 (28)	7 (19)	14 (37)
ORR	65 (88)	33 (92)	32 (84)
			*P* > 0.99
Not recorded	3 (4)	0	2 (5)
			
2D standard response (EOT), *n* (%)			
PD	8 (11)	8 (22)	4 (11)
SD	0	0	0
PR	21 (28)	11 (31)	10 (26)
CR	35 (47)	14 (39)	21 (55)
ORR	56 (77)	25 (69)	31 (82)
			*P* = 0.3
Not recorded	11 (15)	3 (9)	2 (5)
			
IELSG score risk group, *n* (%)			
Low (0-1)	15 (20)	8 (22)	7 (18)
Intermediate (2-5)	59 (80)	28 (78)	31 (82)
			*P* = 0.8
			
2-year PFS, %		61 (95% CI 43-75)	59 (95% CI 41-73)
			*P* > 0.99
2-year OS, %		71 (95% CI 53-84)	70 (95% CI 51-83)
			*P* > 0.99

DLBCL, diffuse-large B-cell lymphomal; ECOG, Eastern Cooperative Oncology Group; LDH, lactate dehydrogenase; R-MPV, rituximab, methotrexate, procarbazine, vincristine; MATRIX, methotrexate, cytarabine, thiotepa, rituximab; RT, radiation therapy; ASCT, autologous stem cell transplant; 2D, 2-dimensional; PD, progression disease; SD, stable disease; PR, partial response; CR, complete response; IELSG, International Extranodal Lymphoma Study Group.

All eligible patients had an evaluable baseline MRI, 65 had an evaluable interim MRI, and 54 had an evaluable EOT MRI. In total, 193 MRIs were analyzed for this study.

### 3D Tumour Volumes

The distribution of baseline MRI volumes is skewed (Kolmogorov–Smirnov test, *P* < 0.001) with a coefficient of variation of 1.64 (Figure 2A). Seven observations were considered outliers (cutoff: 51 ml using 1.5× interquartile range). Baseline median volume was 11.8 ml (range: 0.13-331.6), interim median volume was 1 ml (range: 0-57.05). Median i3DVR and EOT 3DVR was 10 ml (range:0.13-331.6), and 10.2 ml (range:0.13-331.6), respectively.

### *Baseline 3DV, Patient Characteristics*, *and Survival Outcomes*

**Figure 3.**
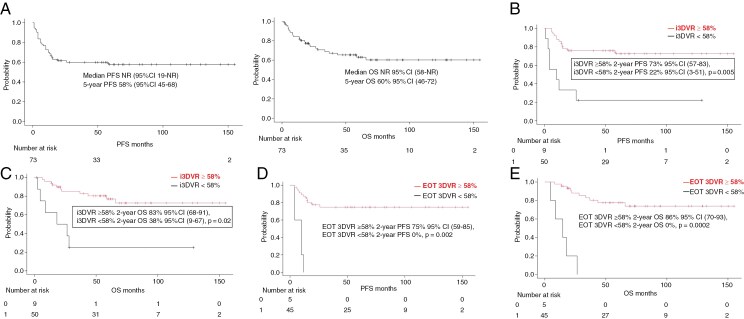
Survival Outcomes for the Whole Cohort and According to i3DVR ≥ 58% at Interim and End of Treatment Time-points. (A) 5-year PFS and 5-year OS for the whole cohort. (B) PFS of patients with i3DVR ≥ 58% and < 58%. (C) OS of patients with i3DVR ≥ 58% and < 58%. (D) PFS of patients with EOT 3DVR ≥ 58% and < 58%. (E) OS of patients with EOT 3DVR ≥ 58% and < 58%. I3DVR, interim 3-dimension volume reduction; PFS, progression-free survival; OS, overall survival; EOT, end of treatment; CI, confidence interval.

Median follow up was 61.9 months (range: 1.9-155.1). Median PFS and OS were not reached in the whole cohort with 5-year PFS and 5-year OS of 58% (95%CI 45-68) and 60% (95%CI 46-72), respectively (Figure 3A).

No difference in PFS or OS between patients divided by using a median binary 3DV cutoff with baseline 3DV more than 11.8 ml compared to baseline 3DV less than 11.8 ml was observed (*P* > 0.99) (Supplementary Table 1). No significant differences in PFS and OS were observed when using quartile (7.25 ml, 28.67 ml) or tertiles (9.37 ml, 24.705 ml) as the cutoff. Moreover, when comparing the upper and lower quartiles or tertiles, no significant difference in PFS and OS was observed in our cohort (*P* = 0.63 and *P* = 0.78, respectively, data not shown). No association with PFS and OS in patients with i3DV less then median i3DV (1 ml) was observed (Supplementary Table 1).

### Interim and EOT Standard and 3DV Tumour Response Association with Survival

Standard 2D response criteria measured at the interim and EOT time-points demonstrated that patients who achieved only an SD or PD on either interim or EOT scans had significantly shorter PFS and OS (Supplementary Table 1) than those with CR or PR. However, among all responding patients (ie CR or PR using standard 2D criteria), achieving a CR did not confer statistically significantly superior PFS or OS compared to PR at either the interim assessment or EOT (*P* > 0.05, Table 2).

**Table 2.** Distribution of Baseline Characteristics between Comparable Groups and Outcomes

	i3DVR ≥ 58%	i3DVR < 58%	EOT 3DVR ≥ 58%	EOT 3DVR < 58%	iCR	iPR	EOT CR	EOT PR
Age ≥ 60 years, % (*n*/*N*)	67 (34/51)	75 (6/8)	68 (32/47)	60 (6/10)	62 (13/21)	68 (30/44)	71 (24/34)	62 (13/21)
Age < 60 years, % (*n*/*N*)	33 (17/51)	25 (2/8)	32 (15/47)	40 (4/10)	33 (7/21)	32 (14/44)	29 (10/34)	38 (8/21)
*P*	>0.99		0.7		>0.99		0.6	
Male, % (*n*/*N*)	56 (33/51)	3/8	60 (28/47)	40 (4/10)	67 (14/21)	59 (26/44)	62 (21/34)	57 (12/21)
Female,% (*n*/*N*)	35 (18/51)	63 (5/8)	40 (19/47)	60 (6/10)	33 (7/21)	41 (18/44)	38 (13/34)	43 (9/21)
*P*	0.2		0.3		0.6		0.8	
LDH > ULN, % (*n*/*N*)	8 (4/48)	12.5 (1/8)	15 (6/41)	20 (2/10)	0	14 (6/42)	3 (1/30)	20 4/20
LDH <= ULN, % (*n*/*N*)	92 (44/48)	88 (7/8)	85 (35/41)	80 (8/10)	100 (18/18)	85 (36/42)	97 (29/30)	80 (16/20)
*P*	0.6		0.6		0.2		0.1	
ECOG 0-1, % (*n*/*N*)	67 (34/51)	63 (5/8)	65 (31/47)	60 (6/10)	57 (12/21)	70 (31/44)	32 (11/34)	38 (8/21)
ECOG 2-4, % (*n*/*N*)	33 (17/51)	38 (3/8)	34 (16/47)	40 (4/10)	43 (9/21)	30 (13/44)	68 (23/34)	62 (13/21)
*P*	>0.99		0.7		0.4		0.8	
Deep brain structures involvement, % (*n*/*N*)	69 (35/51)	38 (3/8)	83 (39/47)	70 (7/10)	90 (19/21)	64 (28/44)	74 (25/34)	71 (15/21)
No deep brain structures involvement, % (*n*/*N*)	25 (13/51)	63 (5/8)	17 (8/47)	30 (3/10)	10 (2/21)	36 (16/44)	26 (9/34)	29 (6/21)
*P*	0.1		0.4		0.04		>0.99	
IESLG score 0-1, % (*n*/*N*)	20 (10/51)	25 (2/8)	15 (7/47)	40 (4/10)	10 (2/21)	23 (10/44)	18 (6/34)	10 (2/21)
IESLG score 2-5, % (*n*/*N*)	80 (41/51)	75 (6/8)	85 (40/47)	60 (6/10)	90 (19/21)	77 (34/44)	82 (28/34)	90 (19/21)
*P*	0.7		0.09		0.3		0.7	
CSF protein > ULN, % (*n*/*N*)	17 (7/42)	29 (2/7)	53 (20/38)	20 (2/10)	63 (10/16)	47 (18/38)	54 (14/26)	74 (14/19)
CSF protein <= ULN, % (*n*/*N*)	83 (35/42)	71 (5/7)	47 (18/38)	80 (8/10)	38 (6/16)	53 (20/38)	46 (12/26)	26 (5/19)
*P*	0.8		0.08		0.4		0.2	
Baseline 3DV ≥ 11.8 ml, % (*n*/*N*)	47 (24/51)	62 (5/8)	40 (19/47)	70 (7/10)	33 (7/21)	59 (26/44)	41 (14/34)	52 (11/21)
Baseline 3DV < 11.8 ml, % (*n*/*N*)	53 (27/51)	38 (3/8)	60 (28/47)	30 (3/10)	67 (14/21)	41 (18/44)	59 (20/34)	48 (10/21)
*P*	0.5		0.2		0.07		0.6	
2-year PFS, % (95%CI)					81 (56-92)	61 (45-73)	84 (59-90)	62 (38-79)
*P*					0.2		0.06	
2-year OS, % (95%CI)					88 (61-97)	71 (55-82)	86 (67-95)	76 (51-89)
*P*					0.35		0.07	

PFS, progression-free survival; OS, overall survival; ECOG, Eastern Cooperative Oncology Group; LDH, lactate dehydrogenase; ULN, upper normal limit; IELSG, International Extranodal Lymphoma Study Group; CSF, cerebrospinal fluid; 3DV, 3-dimensional volume; ml, milliliter; EOT, end of treatment; CR, complete response; PR, partial response; iCR, interim complete response, iPR, interim partial; response.

We analyzed the i3DVR and its association with outcomes among patients who achieved a standard criteria interim CR or PR. First, we used the previously published 3DVR cutoff of >65%. Patients with a i3DVR ≥ 65% had 2-year PFS and 2-year OS 72% (95% confidence interval [CI] 57-83) and 83% (95%CI 68-91), respectively, whereas patients with 3DVR < 65% had 2-year PFS and OS of 22% (95%CI 3-51) and 38% (95%CI 9-67), respectively (*P* < 0.001, Supplementary Figure 1A and B). Of note, no difference in PFS and OS was observed when applying the published cutoff of 97% for 3DVR in PCNSL (median PFS and OS were not reached (NR), *P* > 0.99), (Supplementary Figure 1C and D).

The ROC analysis for our cohort revealed 56% for PFS (AUC 0.61) and 58% (AUC 0.62) for OS as the clinically optimal 3DVR cutoffs and we used 58% for our survival analysis. Using this ROC-determined cut off, as expected, the associations with 3DVR were similar to that when using the published 3DVR cutoff of 65% above. Patients with i3DVR ≥ 58% compared with i3DVR < 58% had better 2-year PFS (73% (95%CI 57-83) versus 22% (95%CI 3-51), *P* < 0.05) and 2-year OS (83% (95%CI 68-91) versus 38% (95%CI 9-67), respectively, *P* < 0.05, see Figure 3B and C). Additionally, those who achieved EOT 3DVR ≥ 58% from baseline volume compared with EOT 3DVR < 58% had longer PFS and OS (2-year PFS 75% (95%CI 59-85) versus 0% and 86% 95%CI 70-93 versus 0%, respectively, *P* < 0.001, see Figure 3D and E). We additionally analyzed the frequency of iCR and i3DVR ≥ 58% after 2 and 5 cycles of treatment and there was no difference in the number of iCR and i3DVR ≥ 58% after 2 and 5 cycles, respectively (Supplementary Table 2).

No significant differences were observed in the distribution of baseline characteristics as age ≥ 60 (yes/no), gender (male/female), ECOG (0-1/2-4), LDH > upper normal limit (ULN) (yes/no), IELSG score (low risk 0-1/intermediate and high risk 2-5) and level of protein in CSF (normal/elevated) between patients with baseline 3DV ≥ 11.8 ml and <11.8 ml, responding patients with i3DVR ≥ 58% and <58%, EOT 3DVR ≥ 58% and <58%, who achieved standard 2D-assessed interim and EOT CR compared to PR at the respective timepoint. The involvement of deep brain structures was more frequently seen in patients who achieved interim CR, but no difference was noted between patients with EOT CR and PR, i3DVR ≥ 58% and <58%, EOT 3DVR ≥ 58% and <58% (Table 2). In the univariate analysis no association between outcomes and involvement of deep brain structures and IELSG score was observed (Table 3).

**Table 3.** Median and 12-months PFS and OS Depending on Baseline Characteristics

	Median PFS, months	12-months PFS, %	*P*	Median OS, months	12-months OS, %	*P*
Age ≥ 60 years	NR (95%CI 15-NR)	69 (95%CI 54-80)	0.6	NR (95%CI 58-NR)	83(95%CI 69-91)	0.9
Age < 60 years	NR (95%CI 12-NR)	71 (95%CI 48-85)		NR (95%CI 21-NR)	79(95%CI 57-90)	
Male	NR (95%CI 19-NR)	71 (95%CI 55-82)	0.6	NR (95%CI 58-NR)	83(95%CI 68-92)	0.7
Female	NR (95%CI 12-NR)	68 (95%CI 48-81)		NR (95%CI 26-NR)	80 (95%CI 61-91)	
LDH > ULN	7.5 (95%CI 1-NR)	25 (95%CI 4-56)	0.01	33 (95%CI 6-NR)	57(95%CI 17-83)	0.2
LDH <= ULN	NR (95%CI 58-NR)	72 (95%CI 59-82)		NR (95%CI 58-NR)	84(95%CI 72-92)	
ECOG 0-1	NR (95%CI 14-NR)	70 (95%CI 54-80)	0.8	NR (95%CI 37-NR)	85(95%CI 71-93)	0.6
ECOG 2-4	NR (95%CI 12-NR)	64 (95%CI 42-79)		NR (95%CI 26-NR)	76(95%CI 54-88)	
Deep brain structures involvement	NR (95%CI 15-NR)	74 (95%CI 60-83)	0.3	NR (95%CI 66-NR)	85(95%CI 72-92)	0.1
No deep brain structures involvement	58 (95%CI 6-NR)	53 (95%CI 28-72)		58 (95%CI 15-NR)	74 (95%CI 48-88)	
IESLG score 0-1	26 (95%CI 2-NR)	44 (95%CI 19-68)	0.2	28 (95%CI 8-NR)	71 (95%CI 40-88)	0.1
IESLG score 2-5	NR (95%CI 19-NR)	74 (95%CI 61-84)		NR (95%CI 66-NR)	84 (95%CI 72-92)	
CSF protein > ULN	NR (95%CI 8-NR)	77 (95%CI 57-88)	0.04	NR (95%CI 44-NR)	83 (95%CI 65-93)	
CSF protein <= ULN	15 (95%CI 8-NR)	55 (95%CI 36-70)		66 (95%CI 18-NR)	79(95%CI 60-90)	0.3
Baseline 3DV ≥ 11.8 ml	NR (95%CI 15-NR)	64 (95%CI 46-77)	0.8	NR (95%CI 34-NR)	78 (95%CI 60-88)	0.9
Baseline 3DV ≥ 11.8 ml	NR (95%CI 10-NR)	73 (95%CI 56-84)		NR (95%CI 37-NR)	86 (95%CI 69-93)	

PFS, progression free survival; OS, overall survival; ECOG, Eastern Cooperative Oncology Group; LDH, lactate dehydrogenase; ULN, upper normal limit; IELSG, International Extranodal Lymphoma Study Group; CSF, cerebrospinal fluid; 3DV, 3-dimensional volume; ml, milliliter; CI, confidence interval; NR, not reached.

## Discussion

Our analysis of semiautomated MRI 3DV measurements at baseline, interim, and EOT time-points evaluates patients with PCNSL undergoing curative-intent CIT, with a uniquely long follow up period. We described a wide range of baseline tumor volumes at diagnosis (range 0.13-331 ml, median 11 ml) with a high coefficient of variation. This volume distribution and median are consistent with other published PCNSL studies.^14,17^ More importantly for the first time we demonstrated the role of 3DVR and its association with outcomes at both interim and EOT time-points in responding PCNSL patients. Almost two-thirds of patients with substantial 3DVR at early interim response assessment have no relapse or disease progression with follow up while the median PFS of responding according to standard criteria patients with 3DVR < 58% is less than one year. In contrast, although we identified an association between inferior outcomes and PD or SD compared to responding disease using standard 2D IPCG response assessment, no difference was observed between 2D-assessed CR versus PR and outcomes in our cohort, consistent with previous studies.^12–14,19^

These findings are similar to a recent study by Lauer et al.^16^ however there are noteworthy differences in methodology and results. Of significance, their significantly higher reported 3DVR cutoff of 97% could not be validated in our cohort. This may have been due to our considerably longer patient follow up median follow up of 61.9 months versus the Laurer’s study 20.9 months, or the fact we only included treatment-naïve patients with diffuse-large B-cell lymphoma histology. This contrasts the Lauer analysis which included secondary relapses plus a broader range of lymphoma histology. Furthermore, their work featured a heterogeneously treated patient cohort wherein 48% of patients received consolidation with autologous stem cell therapy (ASCT), whereas in our study cohort only 23% (17/74) received consolidation RT and 4% (3/74) underwent ASCT.

The difficulty in differentiating true contrast-enhancing disease and expected postoperative changes in enhancement are acknowledged by the current IPCG guidelines. Additionally, they acknowledge the limitations of 2D tumor burden assessment due to interobserver variability and certain distributions of disease common in PCNSL, such as heterogenous or infiltrative disease.^9^ Our data support the guideline suggestion that volumetric analysis improves the reliability and reproducibility of burden of disease assessment. This was the case for our cohort whether we used our ROC defined 58% 3DVR or the previously published 65% used by GBM studies.

Emerging data in primary brain tumors, brain metastases, and lymphoma also support the superiority of 3DV-based methods in clinical utility and reliability in increasing interobserver agreement.^11,13,15,17^ Various approaches for calculating 3D volumetric response of brain tumors are being explored in the wider literature. Regarding primary brain tumors, Ellingson et al. used contrast-enhancing T1 subtraction maps to measure 3D volumetric response, demonstrating a significant association with OS in patients treated with cabozantinib in relapsed GBM.^15^ To contrast, Gahrmann et al. used a variety of volumetric techniques, analyzing both nonenhancing tumor and enhancing tumor volume.^20,21^ It was initially found 3D volumetric methods were equivalent to 2D criteria for predicting PFS. However, a subsequent study was conducted which found improved prediction of adverse outcomes by using a lower threshold (25%) for volume increase at early interval follow-up (6 and 12 weeks), but a high rate of early progression precluded statistical differentiation between 2D and 3D based methods. These techniques are now being explored in PNCSL given the inherent difficulty in determining tumor size in this disease.

The unpredictable clinical course of PCNSL necessitates the discovery of new biomarkers (in addition to 3D MRI) that could revolutionize its management. Improvements in outcome prediction would spare many patients from significant treatment toxicity. Response assessment is currently limited to 2D lesion assessment, retinal disease, and CSF cytology. Given the clear limitations, other biomarkers are being explored which leverage recent technological breakthroughs. CSF cell-free DNA (cfDNA) is one such emerging biomarker. Recent studies^22,23^ have demonstrated that baseline CSF cfDNA burden predicted significantly worse survival. Further development is required to increase detection efficiency and determine how different mutation profiles respond to treatment. Functional molecular imaging (PET, combined PET-MR) is pivotal in systemic lymphoma but more limited in PCNSL due to tracer accumulation in the brain. Current studies are examining indices, use of machine learning to analyze the imaging data and alternative radiotracers to improve its diagnostic and predictive utility.^24^ Prospective studies that analyze a combination of these newer biomarkers will be of significant value in improving our understanding of the course and treatment of this disease.

Our study had limitations inherent to retrospective studies including some heterogeneity in patient selection, treatment delivery, interim imaging time-points and the lack of paired scans at all time-points for all patients. Missing data, and the influence of dose reductions, dose delays and toxicity, which we were unable to accurately collect also contributed to the limits of this study. Furthermore, the method used for 3DV measurement has not been previously validated for lymphoma, so prospective validation in a uniformly treated population is required. As a first step, we are in the process of validating these results in the prospective national Australasian Leukaemia and Lymphoma Group “BLOCK CNSL” study (ACTRN12619000518167).

## Conclusion

In summary, our study demonstrates that in PCNSL semiautomated interim and EOT 3DVR are associated with survival outcomes in patients receiving first-line CIT whereas an association with outcome and standard 2D CR versus PR was not observed. Interim 3DVR may identify nonresponders who should be considered for clinical trials. Based on our findings, further prospective validation is underway, and future routine incorporation of 3DV assessment should be considered to improve prognostication and response assessment of PCNSL.

## Supplementary Material

vdaf090_suppl_Supplementary_Tables_S1-S2_Figure_S1

## Data Availability

The data that support the findings of this study are available from the corresponding author upon reasonable request.
