# Supplementary material for: Refinement of primary central nervous system lymphoma prognostication and response assessment using 3-dimensional MRI
Source: Neurooncol Adv. 2025 May 5;7(1):vdaf090. doi: 10.1093/noajnl/vdaf090 (PMC12202003; doi:10.1093/noajnl/vdaf090)
Supplement: vdaf090_suppl_Supplementary_Tables_S1-S2_Figure_S1 [file vdaf090_suppl_supplementary_tables_s1-s2_figure_s1.docx]

Supplemental figure 1

PFS and OS outcomes according to i3DVR

A PFS of patients with i3DVR ≥65% and <65%

B OS of patients with i3DVR ≥65% and <65%

C PFS of patients with i3DVR≥97% and i3DVR <97%;

D OS of patients with i3DVR≥97% and i3DVR <97%;


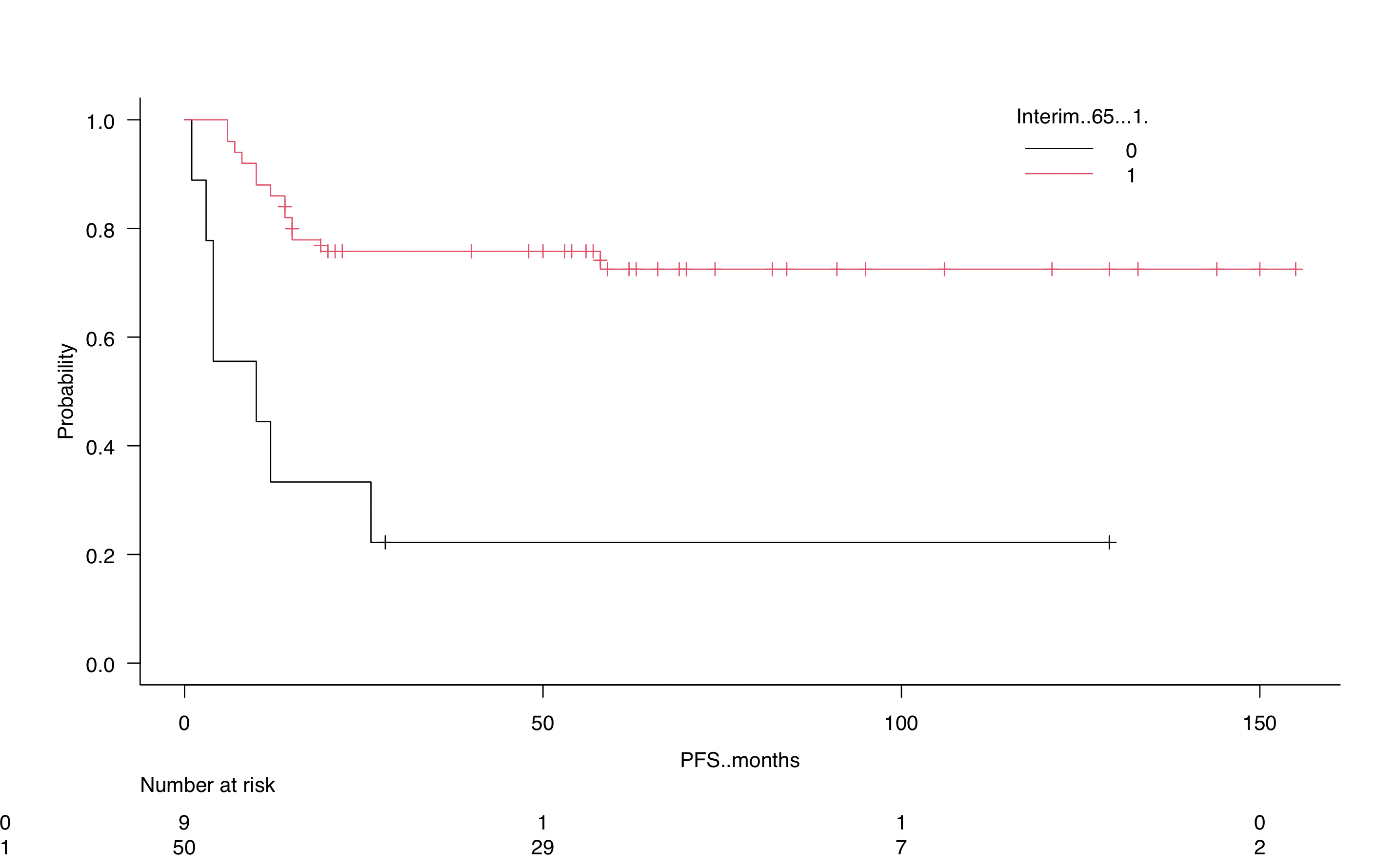


i3DVR ≥65% 2-year PFS 72% (95%CI 57-83),

i3DVR <65% 2-year PFS 22% (95%CI 3-51), p= 0.007

**Interim 3DVR ≥ 65%**

Interim 3DVR < 65%

A


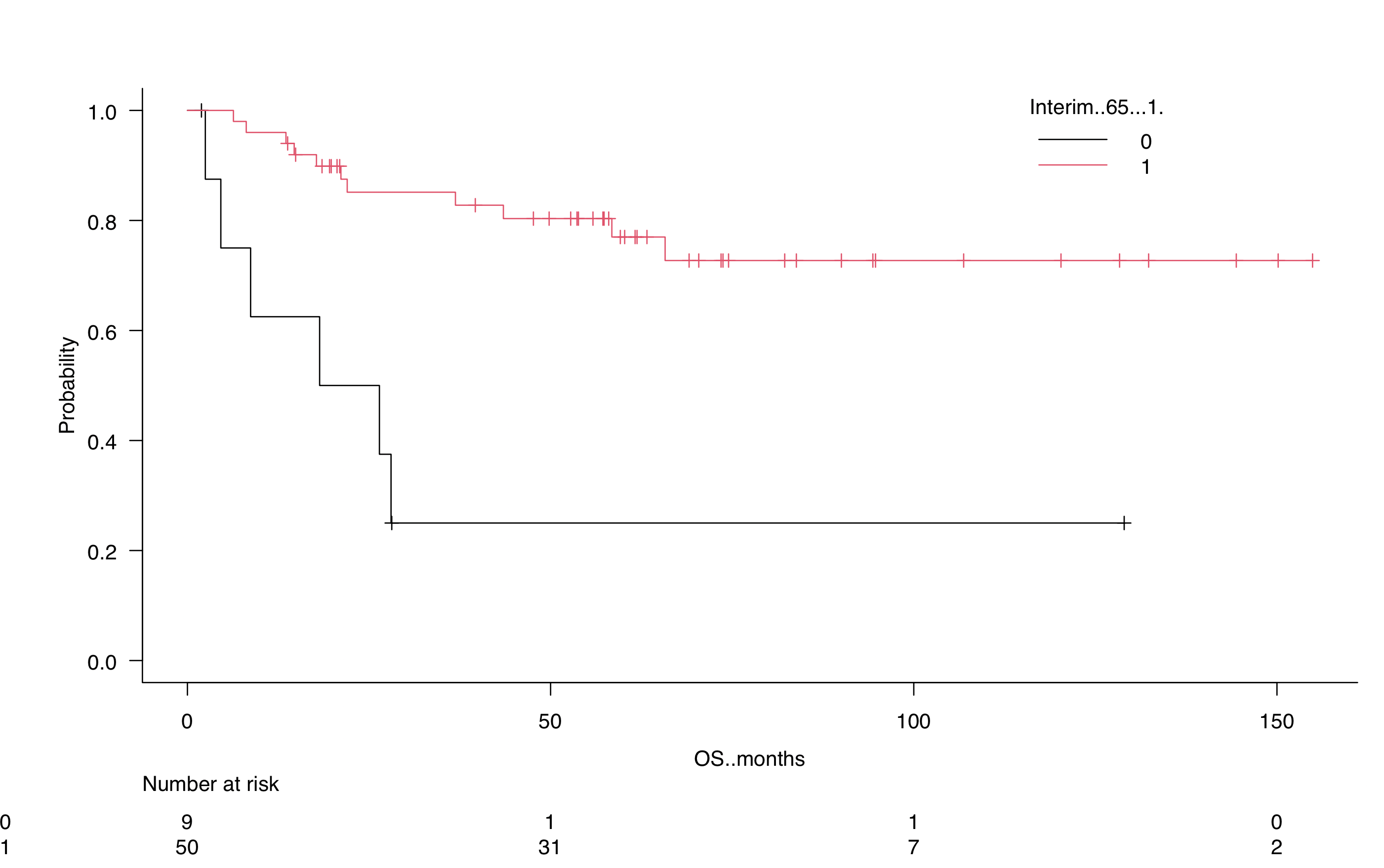


i3DVR ≥65% 2-year OS 83% (95%CI 68-91),

i3DVR <65% 2-year OS 38% (95%CI 9-67), p= 0.01

**i3DVR ≥ 65%**

i3DVR < 65%

B


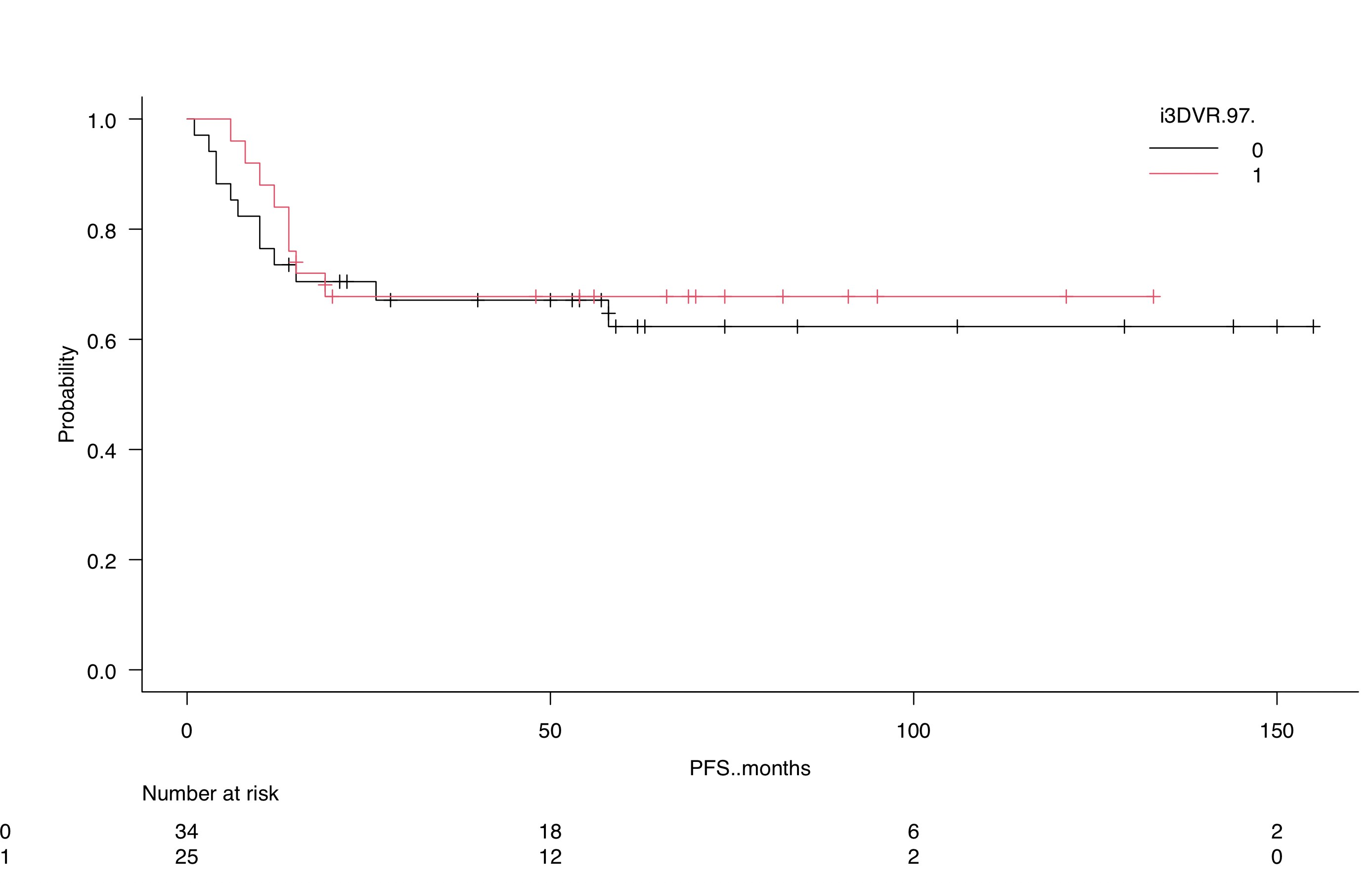


**i3DVR ≥ 97%**

i3DVR < 97%

i3DVR ≥97% 2-year PFS 68% (95%CI 46-82),

i3DV 3DVR <97% 2-year PFS 67% (95%CI 48-80), p>0.99

C


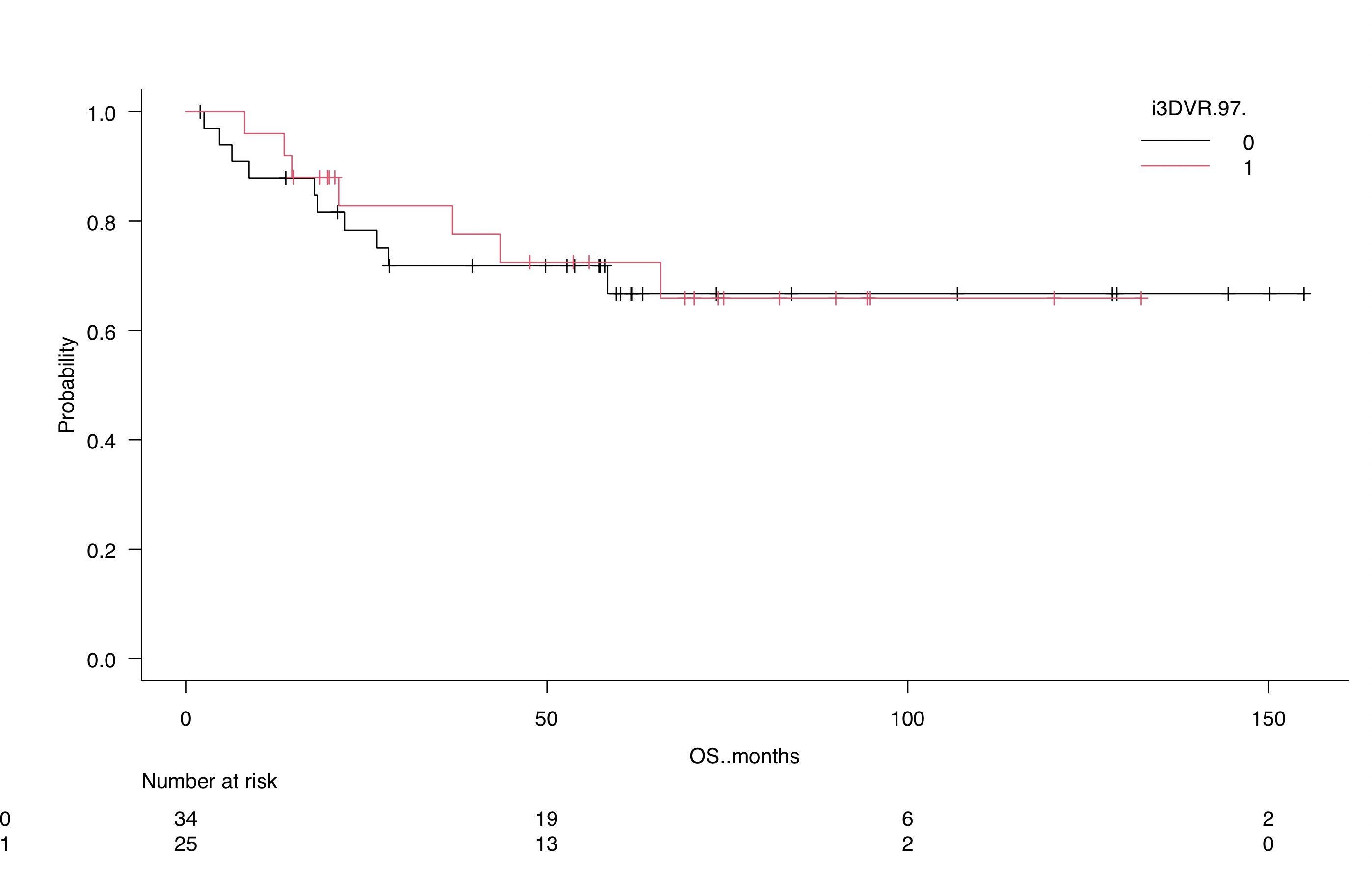


**Interim 3DVR ≥ 97%**

Interim 3DVR < 97%

i3DVR ≥97% 2-year OS 78% (95%CI 54-90),

i3DV <97% 2-year OS 75% (95%CI 56-87), p>0.99

D

I3DVR – interim 3-dimensional volume reduction, PFS – progression free survival, OS – overall survival, NR – not reached, CI – confidence interval

Supplemental Table 1

Patient’s outcomes according to interim and EOT standard 2D response and interim 3DV

|  | 2-year PFS, % | 2-year OS, % |
| --- | --- | --- |
| Interim SD/PD | 0 | 0 |
| Interim PR/CR | 69 (95%CI 56-79) | 79 (95%CI 66-88) |
| p | 0.0006 | 0.008 |
|  |  |  |
| EOT SD/PD | 77 (95%CI 63-86) | 0 |
| EOT PR/CR | 0 | 84 (95%CI 71-92) |
| p | 0.00004 | 0.00009 |
|  |  |  |
| i3DV ≥1 ml | 60 (95%CI 43-75) | 66 (95%CI 45-80) |
| i3DV <1 ml | 59 (95%CI 41-73) | 77 (95%CI 57-88) |
| p | 0.2 | 0.6 |
|  |  |  |
| 3DV ≥1.8 ml | (95%CI 43-75) | (95%CI 45-80) |
| 3DV <1.8 ml | (95%CI 41-73) | (95%CI 57-88) |
| p | >0.99 | >0.99 |

I3DV – interim 3-dimensional volume, CI – confidence interval, PFS – progression free survival, OS – overall survival, PR – partial response, CR – complete response, CI – confidence interval

Supplemental Table 2

Distribution of iCR and i3DVR≥58% after 2 and 5 cycle of therapy

|  | N | iCR n (%) | i3DVR≥58% n (%) |
| --- | --- | --- | --- |
| After 2 cycles | 31 | 7 (21.9) | 19 (59.4) |
| After 5 cycles | 43 | 14 (32.6) | 31 (72.1) |
| p | 0.07 | 0.44 | 0.45 |

IPCG - International PCNSL Collaborative Group, i3DVR – interim 3-dimensional volume reduction, iCR- interim complete response
